# The American Medical Press

**Published:** 1898-09

**Authors:** 


### THE AMERICAN MEDICAL PRESS.

The average busy practitioner—and even he who is not so blessed —rarely gives a thought to the Medical Press of this country, to what it is, to what it costs in effort and money, to the immense power for good which lies latent in it, and for progress or deterioration of which he alone is directly responsible. If he thinks of it at all, he soon dismisses it as a necessity—agreeable or otherwise, as he happens to be fond of his profession or is merely a money-grabber—for which he pays or expects to pay in the very distant future. A short dissertation on this subject, therefore, will be a very profitable employment of time to most of our esteemed readers, and will be the medium of information upon a topic than which none can be of greater interest, because this touches most closely the physician himself in his reputation and in his capacity and the opportunity for earning his daily bread. For the medical press is what its subscribers make it. It is a lever of mighty power—"a lever to move the world"—and its significance to the profession, is whether the handle of this lever is controlled by the profession or whether the profession rest upon the short end and the long end is held in the hands of those who know its value and have so long exploited it. The value of the press to us—and who will deny its potential might?—lies in its control, its universal and absolute control, by ourselves, by medical men who are bound by professional obligations, by that common interest which binds us all, that remnant of medical ethical feeling which, notwithstanding the stupendous and blind selfishness of the individual practitioner, is still powerful enough to define our conduct, each to all and all to each, within certain fixed limits. Encourage with your support such a press, make it an accomplished fact by subscribing and *promptly paying your subscriptions,* and you need not doubt that the editors and medical proprietors will unite and act together for the common good. Medical editors know very well that a powerful press must be a united press, and must equally represent a powerful and united body of men—*i. e.*, the profession; for if we editors had universal influence and our written words the convincing force of a Cicero and a Demosthenes, of what avail if the profession which we represented and for which we spoke remained disrupted, weak and incapable of self-government and the use of power? The profession and its press are indissolubly united for good or evil. Reform your press, and it will in turn reform and unite you. Support and encourage medical proprietorship in your journalism—not by platonic good wishes, but by your *exclusive* patronage and by your money, and you will find the lever of a mighty press for use at your hand. Encourage, by your selfish indifference to everything which may benefit others as well as your individual self, journalistic proprietorship by lay publishing houses, and your medical press will continue, as it has hitherto done, to *use you.*

When this journal first entered the field, something more than six years ago, it was almost the only first-class journal owned and edited by a professional man in this country; to-day there are probably a dozen or more in this category. We doubt not that at least a majority of these medical proprietors and editors are actuated by singleness of purpose to work for the good of the profession, to create a great press, and that they are sustained by the hope that the profession will one day recognize practically the honesty of their purpose and its self-sacrifice. Do you fancy that all these journals are supported by the profession? Does that fatuous thought, perchance, cross the mind of the subscriber who throws his bill aside to be paid in a year or two? Does that contributor also believe this who sends his article to be published by some lay medical journal rather than by one owned by a medical man, not because the former is better or has a larger circulation (thank God, that excuse will not hold to-day), but because he thinks he can get a longer subscription credit and better terms for new medical books?

Remember this: If you wish this movement toward bringing the medical press under medical control to succeed, *you must not*

*expect credit; you must pay your subscriptions in advance.* You must make up your mind to that sacrifice for the sake of the great good to be obtained. Medical proprietors have not large capital behind them; *you* must supply the capital to work with. After all, you are only expected to pay for what you receive, and we say, without a moment's hesitation, in the case of every journal for which you may have subscribed, you get your money's worth and more. If, on the other hand, you have no sympathy with the struggles of these medical men to give you a medical press which you yourselves shall own and whose policy shall always be dictated by your interests; if you prefer your press to remain always merely a grab-bag from which you will have the privilege, as you wish, to draw out a more or less interesting original article or society proceeding, you can accomplish this end without exertion. It is only necessary to neglect to pay your subscription. You will force, in this gentle and easy manner, every medical proprietor out of the journalistic field, and you will hand over the medical press absolutely to the great lay publishing houses who do not expect you to support their journals, which are not published in your interests but as advertising mediums for their other publications.

It is this uneven fight which we editors are fighting, and the question at stake is whether the profession to which we belong and in whose interest we work mean to aid us or to turn aside. After all, it is for you, the profession, not for us; for we venture to say there is not one medical proprietor in this country who could not earn more at his profession with half the labor expended in journalism than he could ever expect from the most generous support his subscribers could give him. The decision and the responsibility lie with you. It is easy (for the individual sacrifice is not great) to create a great American medical press under medical control. It is easier still to place it back again where for so many years it remained—on a plane of mediocrity and in the hands of lay publishing houses.—*American Gynecological and Surgical Journal.*

